# Supplementary material for: Prefrontal attentional saccades explore space rhythmically
Source: Nat Commun. 2020 Feb 17;11:925. doi: 10.1038/s41467-020-14649-7 (PMC7026397; doi:10.1038/s41467-020-14649-7)
Supplement: Supplementary file 1 — Supplementary Information [file 41467_2020_14649_MOESM1_ESM.pdf]

Supplementary information

Supplementary Note 1: Description of the neuronal population response properties

The recorded receptive fields are quite large, as typically described in the FEF. Sixty-one percent of the MUA channels had a significant ***target related*** response on correct trials. Of these, 23.5% of the recorded RFs encompass one visual quadrant, 24.2% encompass two ipsilateral visual quadrants, 4.6% encompass two opposing visual quadrants, 21.6% encompass three visual quadrants and 26.1% encompass 4 visual quadrants. Seventy-three percent of the MUA channels had a significant ***attention related*** response on correct trials. Of these, 14.1% of the recorded RFs encompass one visual quadrant, 14.4% encompass two ipsilateral visual quadrants, 3.6% encompass two opposing visual quadrants, 20.2% encompass three visual quadrants and 47.7% encompass 4 visual quadrants. This diverse receptive field structure of the data was critical for the success of the linear decoding approach that we are using here. Noteworthy is the fact that, in addition to significantly modulated neurons, non-significantly modulated neurons also contributed to the decoder[1]. Supplementary fig. 2 further reports the MUA spatial attention selectivity on an exemplar MUA signal, an exemplar session and across recording sessions.

Supplementary Note 2: Averaging filter impact on decoded signal frequency content

Independent time series of our data (for each channel and each trial) are averaged over 50ms time windows (moving averaging filter of frequency fa=20Hz) at every 1ms time step (sampling frequency fs=1kHz). This results in a continuous time series (fig. 8). These rhythmic time series can be modelled by artificial attention signals (supplementary fig. 4a), the frequency content of which is strongly attenuated at fs, 2fs, 3fs etc. (supplementary fig. 4d, light gray).

In the decoding procedure described in the present manuscript, the rhythmicity of spatial attention is not only captured by individual channel changes in spiking probability, but also in the population code, due to specific co-activation patterns across the entire population that are captured by the decoding procedure. supplementary fig. 4 models this point. Supplementary fig. 4a (left panel), represents an artificial attentional signal sampling space at 24Hz, in time (x-axis), over multiple trials (y-axis). From one trial to the next, the encoded spatial position of attention is different. The 24Hz frequency is higher than the moving averaging filter central frequency (20Hz). For each trial (n=150), 50 independent times series (representing the spiking activity of 50 independent MUA channels), were generated from the corresponding artificial attentional signal as follows. Each channel was associated with a specific spiking probability that varied as a function of the artificial attention signal (position of attention). For each trial, spatial attention was then decoded from this population (supplementary fig. 4a, right panel). Supplementary fig. 4b (blue) represents such decoded spatial attention information on a representative trial. It captures both frequency specificity and power (supplementary fig. 4c) of the input rhythmic spatial attention signal on the same trial (supplementary fig. 4b, red).

Supplementary fig. 4d generalizes these observations to input rhythmic signals ranging from 3 to 70Hz. The black trace corresponds to the actual input power frequency relationship in our artificial data. The light gray curve represents the recovered frequency content from the moving average filtered input independent time series (fa=20Hz). As predicted by signal processing theory, frequencies at fa and multiples of fa cannot be recovered. The intermediate gray trace represents the frequency content recovered from the population decoding procedure. While this procedure leads to an attenuation of the power of the recovered frequencies that is comparable to that observed on the moving averaging filtered input data, frequencies can be recovered along the entire test frequency range, including at frequencies higher than fa, and at all fa harmonics. This is due to the fact that, the rhythmicity of spatial attention in not only captured by individual channel changes in spiking probability, but also in the population code. While the moving averaging filter cancels fa and fa harmonic frequencies, the 1ms sampling procedure generates specific co-activation patterns across the entire population. These co-activation patterns are captured by the decoding procedure. In our simulations full recovery of fa frequency content is achieved for a number of channels of ten or more (data not shown).

Importantly, all frequency contents reported in the present manuscript are identified against a 95% C.I. generated from random permutation of the data prior to the decoding procedure. As a result, this chance level captures the frequency attenuation described in supplementary fig. 4d.

Supplementary Note 3: Frequency & phase of attentional rhythm across hemispheres

We show that the spiking probability of individual MUA channels is modulated at an alpha-rhythm (fig. 3acd). A very clear alpha-oscillation can also be identified, when the activity of MUAs across all channels is computed for individual trials (super MUA, fig. 3b). This strongly suggests that MUAs from both hemispheres are phase locked. Confirming this prediction, supplementary fig. 6a shows, for an exemplar trial, the super MUA independently computed on the left and right MUA recordings. On this specific trial, both super MUAs oscillated at exactly the same rhythm and are perfectly in-phase. This is confirmed at the level of all trials and all sessions (peak frequency for left mean+/-s.e.=9.72Hz+/- 0.49Hz and right super MUAs mean+/-s.e.=9.8Hz+/- 0.56, Wilcoxon test for equal means, p=0.45; supplementary fig. 6b, across session distribution of phase differences between the left and right super MUA alpha content, circular mean=0.07°, circular s.d.=3.26°). This thus indicates that the spiking rate of the entire FEF population is modulated at a unique rhythm, in-phase between both hemispheres. As discussed in the main text, super MUA rhythmicity reflects an alpha-clocked change in the FEF attention-related information, rather than an information-independent global alpha-modulation. Under the hypothesis that attention is mainly directed to either one hemisphere or the other (at least in the context of the current task), one expects the strict phase locking between the left and right super MUAs to translate into anti-phased left and right attention population contents. In other words, one expects that when attention information content is high in the right cortical population, it is at the same time low in the left cortical population and vice versa. Our current decoding is based on 48-channels. Repeating the cross-temporal decoding analyses presented in fig. 2 using one probe at a time, significantly decreases overall decoding performance (average classification performance of attention allocation: M1: bilateral 43.8%, unilateral 38.1%; M2: bilateral 39.5%, unilateral 33%, absolute chance level at 25%), all the more that our decoding is being performed at a high temporal resolution (50ms). In spite of this drop in overall decoding accuracy, peak oscillatory frequencies identified on the left and right neuronal population decoded attentional signal are indistinguishable (fig. supplementary fig. 6c, p=0.75, Wilcoxon non-parametric test) and similar to the frequencies identified based on bilateral decoding (Wilcoxon non-parametric test, bilateral vs. left, p=0.33; bilateral vs. right, p=0.20). Importantly, the oscillations in the attentional signal between these two left/right neuronal populations are close to anti-phase (supplementary fig. 6d, circular mean=178.28°, circular s.e.=16.24°). Overall, this suggests an interhemispheric coordination in how the attentional spotlight explores visual space between the two hemispheres. This will need to be further explored. Given that the attentional rhythms are indistinguishable whether identified on the left probe, the right probe or both probes, the analyses presented in figures 4, 7 and 8 remain unchanged by this factor. Indeed, these analyses are uniquely driven by the identified attentional rhythms.

Supplementary Note 4: Alpha rhythm in superficial and deeper cortical layers

In our own data, as recordings were performed tangentially to FEF cortical surface, we have no direct assignation of the recorded MUAs to either superficial or deep cortical layers. However, previous studies have shown that pure visual neurons are predominantly located in the supragranular layers of the FEF while visuo-motor neurons are predominantly located in its infragranular layers[4–9]. Pouget et al.[10] further show that supragranular FEF neurons predominantly project to striate visual cortex while infragranular FEF neurons predominantly project to the superior colliculus[11–13]. Interestingly, Buffalo et al.[14] have shown that, in extra-striate area V4, the ratio between the alpha and gamma spike field coherence discriminate between LFP signals in deep (low alpha / gamma spike field coherence ratio) and superficial cortical layers (high alpha / gamma spike field coherence ratio). In Ben hadj Hassen et al.[15], we show that the LFP alpha / gamma spike field coherence ratio provides a very reliable segregation of visual and visuo-motor FEF MUAs at the same recording site. We thus consider that, as has been described for area V4, this LFP alpha / gamma spike field coherence allows for a reliable delineation of superficial and deep layers in area FEF, approximatively defined by a slope of y= 1.4x. In the present study, we did not characterize the oculo-motor properties of the recorded MUA

channels. However, these recordings were performed in exactly the same recording sites as in Ben Hadj Hassen et al.[15]. In the following (supplementary fig. 7a), we thus characterize the alpha / gamma spike field coherence ratio for all of our task-related LFP and segregate them as superficial or deep recording sites, using the same delineation slope as characterized in previous study[15] (see supplementary methods, below).

We then used the same methodology as presented in fig. 3, to quantify the frequency of high spiking probability epochs in superficial and deep FEF layers. Supplementary fig. 7b shows, for a representative trial, alpha filtered super MUA over superficial (red) and deep (blue) channels where alpha frequency amplitude appears higher in the deep (blue) contact. This is confirmed by a more global analysis (supplementary fig. 7c), in which we demonstrate that, across all channels and all sessions high spiking probability epochs expressed a rhythmicity in a strictly comparable alpha frequency range. Importantly, global MUA spiking alpha probability is significantly stronger in the deeper as compared to the superficial cortical layers (Wilcoxon test $p<=0.01$** from 7.5 to 12Hz), possibly suggesting that the alpha clock originates in the deeper cortical layers as reported in numerous studies and various cortical regions[14,16].

Supplementary Note 5: Phase relationship between super MUA and LFPs

The super MUAs reflect the general alpha-clocking of the entire bilateral FEF neuronal population. This activity is phase locked between the two hemispheres (supplementary fig. 6 and related supplementary information). In contrast, individual LFPs reflect both long range inter-areal and local processes on each session. Supplementary fig. 8 represents the frequency content (mean +/- s.e.) of both the task-related LFPs (supplementary fig. 8ab) and the super MUAs (supplementary fig. 8c) across all sessions. LFPs show an enhanced frequency content in the lower theta range (3-5Hz) as well as in the beta frequency range (18-30Hz). These observations are consistent with previous reports[2,3]. In contrast, while the theta peak of the super MUAs is weak, these signals show a marked frequency peak in the alpha range (7-12Hz, coinciding with the frequency range described in the present work, fig. 2 & 3), as well as a consistent peak in the beta frequency range (18-30Hz). Alpha oscillatory mechanisms thus appear to be specific to the super MUAs. Supplementary fig. 8d represents phase-phase coherence between the LFP and super MUA signals. Coherence is enhanced in the three frequency bands identified in supplementary fig. 8ab, namely the lower theta range (3-5Hz), the alpha range (7-12Hz), as well as in the beta frequency range (18-30Hz). Overall, this thus suggests a strong phase coupling between the FEF LFPs and super MUAs. The functional significance of this coupling, its directionality and its causal relationship to attention and perception remains to be explored.

Supplementary Note 6: Classification procedures applied to a dynamic process

Classical classification approaches applied to the decoding of cortical activity are designed to associate specific populational signatures with specific experimental task components or classes. The essence of these approaches is to identify, based on a set of exemplar data (training set), for each class of interest, common response patterns (signal) irrespective of potential inter-trial variability (noise), and to evaluate the efficacy of the identified common response patterns on a novel data set (testing set). In our approach, we push this approach one step further, considering that on each trial, part of the noise (i.e. part of the distance between the observed response pattern and the actual corresponding class response pattern) is actually signal and characterizes attentional dynamics. In other words, we apply machine learning to neuronal signals under the assumption of stability of the attentional spotlight and then we interpret decoding error to the expected class as a signature of attentional dynamics. This is possible because these types of classifiers are driven by both the mean and the variability around the mean of the test class data. The relevance of this approach is confirmed by the fact that, while this might seem counter intuitive, this approach actually proves extremely efficient in capturing both specific neuronal and behavioral processes and accounting for an important part of observed variability (e.g. neuronal response to target and distractor, variations in hit rate, variations in false alarm rates, figures 4 & 7).

Post-cue cross-temporal decoding maps capture the rhythmic nature of attention. This is due to the fact that the cue resets the attentional rhythm. If attention exploration was rhythmic, yet completely random over space, the resultant cross-temporal decoding map wouldn't be expected to show any clear rhythmicity. However, we show that attention expresses predictable exploration patterns between the cued and uncued

quadrants (see fig. 10). These transitions of the attentional spotlight between quadrants fully account for the observed rhythmic oscillations in the post-cue cross-temporal decoding maps.

Supplementary Note 7: Alpha exploration/exploitation model

This model provides a nice comprehensive interpretation of changes in the parieto-frontal neuronal synchronization and coupling properties during 80% validity cued target detection tasks. The task used here is a 100% validity task. There is thus no behavioral drive to explore uncued locations, as one would expect in 80% validity cued target detection tasks. And indeed, the decoded prefrontal attentional spotlight only rarely directly explores the uncued landmarks (fig. 9), though it does visit the different quadrants in a way that varies from one task configuration to another (fig. 10). This important task difference might actually account for the higher alpha content we observe in the decoded spatial attention spotlight traces as compared to the theta content (supplementary fig. 5). In this task configuration, we show that the attentional spotlight explores space at an alpha-clock pace, sometimes visiting the cued location (exploitation) and sometimes visiting uncued spatially irrelevant locations (exploration). Because in our task, exploitation is an unexpected low frequency event, we propose that exploration is the default mode of the system, while exploitation, requires effort or a top-down drive to be implemented. Whether this exploitation is implemented by an independent theta clock remains to be tested. This would reconcile the seemingly contradictory views of the sampling/shifting hypothesis and an alpha exploration/exploitation hypothesis.

Supplementary methods

Oscillations in behavioral performance and reaction time

For Behavioral performance, Hits and Misses from M1 and M2 were cumulated in time (aligned to cue presentation), and merged together across the 19 recording sessions. Behavioral performance, defined as the proportion of (hits/(hits +misses)) was then computed at every millisecond. The spectral analysis of this time series was performed on detrended data using a Morlet Wavelet transform as in Fiebelkorn et al.[2], over the attentional period ranging from 500 ms post cue presentation to 2100ms. Standard error in the power spectrum corresponds to spectral variability during this time interval. Global power spectrum 1/f component was removed from the dataset using a *f normalization (fig. 5). The exact same procedure was used based on reaction times. Reaction times were defined as time between target presentation and manual response (reaction time) were compiled from M1 and M2 across the 19 recording sessions and aligned on cue presentation time.

LFP and super MUA frequency content

LFP power spectrum was estimated for each channel and each trial using Morlet Wavelet transform over an attentional period ranging from 500 ms post cue presentation to 2000ms (1.5s epoch). Global power spectrum 1/f component was removed from the dataset using a *f normalization. Similarly, super MUA power spectrum was estimated for each trial over the same attentional period (1.5s epoch).

Coherency was calculated separately for each hemisphere to avoid possible inter-hemisphere phase effect between LFPs and super MUA. Coherency was calculated using pairwise phase consistency (PPC) on the same 1.5s attentional period epoch than for LFP and super MUA power estimation and based on the fieldtrip ft_connectivityanalysis function[17].

MUA layer attribution

As stated in the result part, our recordings are not tangential to cortical surface. As a proxy to attribute a given recording channel to upper or lower cortical layers we proceeded as follows. For each electrode contact, we estimated, at the time of cue onset in the 4 position attentional task (500-1500 from cue onset), the spike-field coherence in the alpha range (6 to 16 Hz) and the gamma range (40 to 60 Hz). Based on previous literature[14,15] ,we used the ratio between the alpha and gamma spike field-coherence as a proxy to assign the considered LFP signals to a deep cortical layer site (high alpha / gamma spike-field coherence ratio) or to a superficial cortical layer site (low alpha / gamma spike-field coherence ratio). Spike field coherency pairwise phase consistency (ppc) value were computed based on fieldtripREF ft_spiketriggeredspectrum.

In Ben hadj Hassen et al.[15], we show that the LFP alpha / gamma spike field coherence ratio provides a very reliable segregation of visual and visuo-motor FEF MUAs at the same recording site. We thus consider that, as has been described for area V4, this LFP alpha / gamma spike field coherence allows for a reliable delineation of superficial and deep layers in area FEF, approximatively defined by a slope of y= 1.4x. In the present study, we did not characterize the oculo-motor properties of the recorded MUA channels. However, these recordings were performed in exactly the same recording sites as in Ben Hadj Hassen et al.[15]. In the following (supplementary fig. 7a), we thus characterize the alpha / gamma spike field coherence ratio for all of our task-related LFP and segregate them as superficial or deep recording sites, using the same delineation slope as characterized in Ben hadj Hassen et al.[15].

Super MUA phase analyses

For super MUA inter-hemispheric phase comparison, frequency of maximal common power *fm(t)* between left and right super MUA was estimated at each ms time step using cross wavelet transform (in the 7-12Hz alpha frequency range). For each trial of each session, the phase difference *phase_diff(t)* between left and right super MUAs was computed for *fm(t)*. This method thus allows to capture small frequency variations or changes in phase relationship in time.

Influence of 50-ms moving averaging filter on decoded signal

To model the effect of the 50-ms moving averaging filter on the frequency identified in the decoded attentional traces (supplementary fig. 4), we generated an artificial attentional space sampling signal across trial (n=150) and time (1.5s). For each trial (n=150), 50 independent times series (representing the spiking activity of 50 independent MUA channels), were generated from the corresponding artificial input signal as follows to mimic MUA. Each channel was associated with a specific spiking probability that varied as a function of the artificial attention signal (position of attention in time). This 50 channels (population) spiking activity were then decoded using the classical linear approach described in the Neuronal decoding procedure methodological part.

Supplementary references

1. Farbod Kia, S., Åstrand, E., Ibos, G. & Ben Hamed, S. Readout of the intrinsic and extrinsic properties of a stimulus from un-experienced neuronal activities: towards cognitive neuroprostheses. *J. Physiol. Paris* **105**, 115–122 (2011).

2. Fiebelkorn, I. C., Pinsk, M. A. & Kastner, S. A Dynamic Interplay within the Frontoparietal Network Underlies Rhythmic Spatial Attention. *Neuron* **99**, 842-853.e8 (2018).

3. Buschman, T. J. & Miller, E. K. Serial, Covert, Shifts of Attention during Visual Search are Reflected by the Frontal Eye Fields and Correlated with Population Oscillations. *Neuron* **63**, 386–396 (2009).

4. Bruce, C. J. & Goldberg, M. E. Primate frontal eye fields. I. Single neurons discharging before saccades. *J. Neurophysiol.* **53**, 603–635 (1985).

5. Schall, J. D. Neuronal activity related to visually guided saccades in the frontal eye fields of rhesus monkeys: comparison with supplementary eye fields. *J. Neurophysiol.* **66**, 559–579 (1991).

6. Schall, J., Hanes, D., Thompson, K. & King, D. Saccade target selection in frontal eye field of macaque. I. Visual and premovement activation. *J Neurosci* **15**, 6905–6918 (1995).

7. Schall, J. D. & Hanes, D. P. Neural basis of saccade target selection in frontal eye field during visual search. *Nature* **366**, 467–469 (1993).

8. Schall, J. D. & Thompson, K. G. Neural selection and control of visually guided eye movements. *Annu. Rev. Neurosci.* **22**, 241–259 (1999).

9. Goldberg, M. E. & Segraves, M. A. Visuospatial and motor attention in the monkey. *Neuropsychologia* **25**, 107–118 (1987).

10. Pouget, P. *et al.* Visual and motor connectivity and the distribution of calcium-binding proteins in macaque frontal eye field: implications for saccade target selection. *Front Neuroanat* **3**, 2 (2009).

11. Sommer, M. A. & Wurtz, R. H. Composition and topographic organization of signals sent from the frontal eye field to the superior colliculus. *J. Neurophysiol.* **83**, 1979–2001 (2000).

12. Leichnetz, G. R. & Goldberg, M. E. Higher centers concerned with eye movement and visual attention: cerebral cortex and thalamus. *Rev Oculomot Res* **2**, 365–429 (1988).

13. Fries, W. Cortical projections to the superior colliculus in the macaque monkey: a retrograde study using horseradish peroxidase. *J. Comp. Neurol.* **230**, 55–76 (1984).

14. Buffalo, E. A., Fries, P., Landman, R., Buschman, T. J. & Desimone, R. Laminar differences in gamma and alpha coherence in the ventral stream. *PNAS* **108**, 11262–11267 (2011).

15. Hassen, S. B. H., Wardak, C. & Hamed, S. B. Rhythmic variations in prefrontal inter-neuronal correlations, their underlying mechanisms and their behavioral correlates. *bioRxiv* 784850 (2019).

16. Spaak, E., Bonnefond, M., Maier, A., Leopold, D. A. & Jensen, O. Layer-specific entrainment of gamma-band neural activity by the alpha rhythm in monkey visual cortex. *Curr Biol* **22**, 2313–2318 (2012).

17. Oostenveld, R., Fries, P., Maris, E. & Schoffelen, J.-M. FieldTrip: Open source software for advanced analysis of MEG, EEG, and invasive electrophysiological data. *Comput Intell Neurosci* **2011**, 156869 (2011).

Supplementary figures

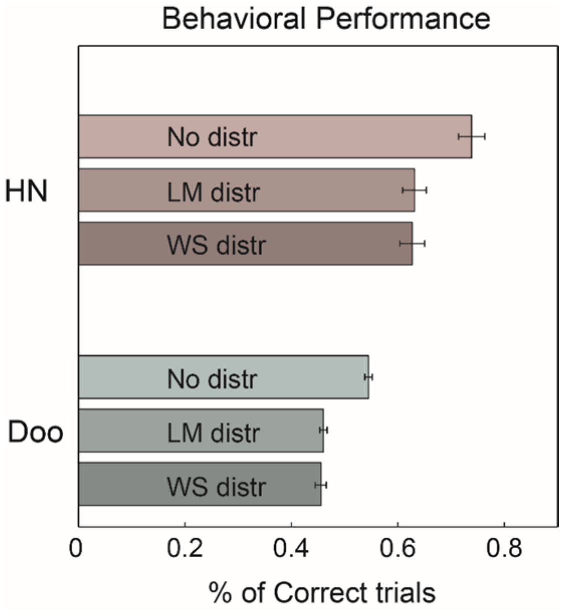

Supplementary Figure 1: ***Behavioral performance of monkeys M1 and M2 for both distractors at the landmark (LM) and distractors in the workspace (WS)***, compared with target detection in the absence (No) of a distractor (median % correct +/- median absolute deviation).

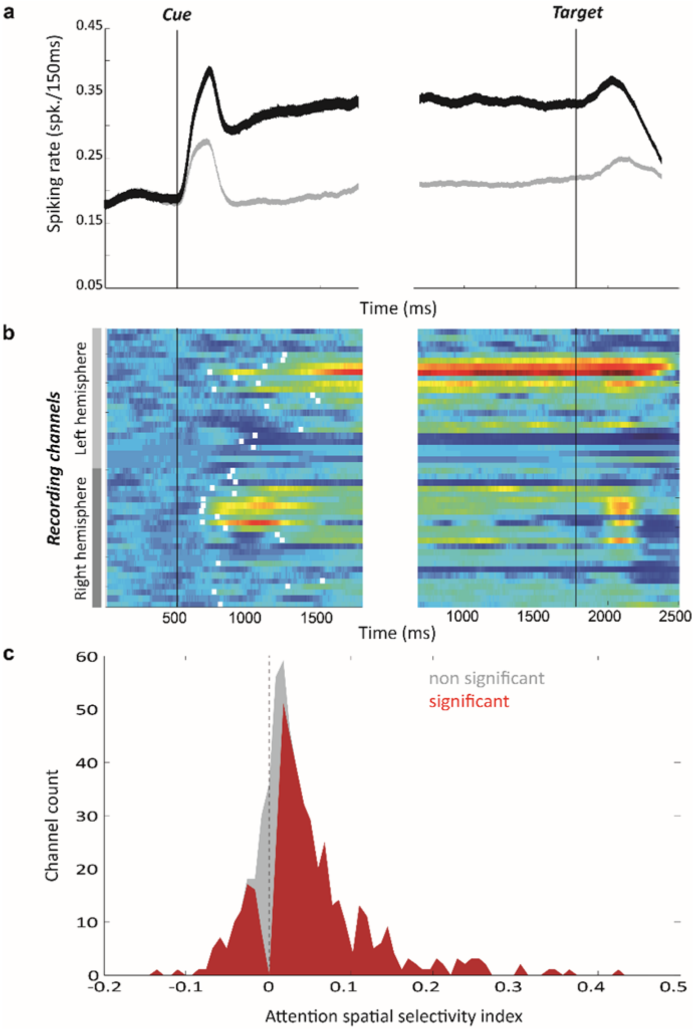

Supplementary Figure 2: ***MUA spatial attention selectivity.*** (a) Single MUA mean (+/- s.e.), when cue is orienting attention towards the preferred (black) or the anti-preferred (gray) spatial location, during the cue to target interval. X-axis represents time around the cue to target interval. (b) MUA spatial attention selectivity for a representative recording session. X-axis represents time around the cue to target interval. Y-axis represents individual channels, separated in left and right hemisphere channels. Each line represents, for each individual channel, the difference between the normalized neuronal response to a cue orienting attention towards the preferred spatial location and the normalized neuronal response to a cue orienting attention towards the anti-preferred spatial location. White ticks represent the onset of statistically significant differences between these two signals (Wilcoxon, $p<0.05$). (c) Distribution of a spatial attention index ((Preferred-AntiPreferred)/(Preferred+AntiPreferred), computed over [-200 0] ms before target onset) across all MUA of all sessions. Red histogram corresponds to channels in which the neuronal activity during this time interval was significantly different between the preferred and the anti-preferred spatial attention responses (Wilcoxon, $p<0.05$, gray, no significant difference).

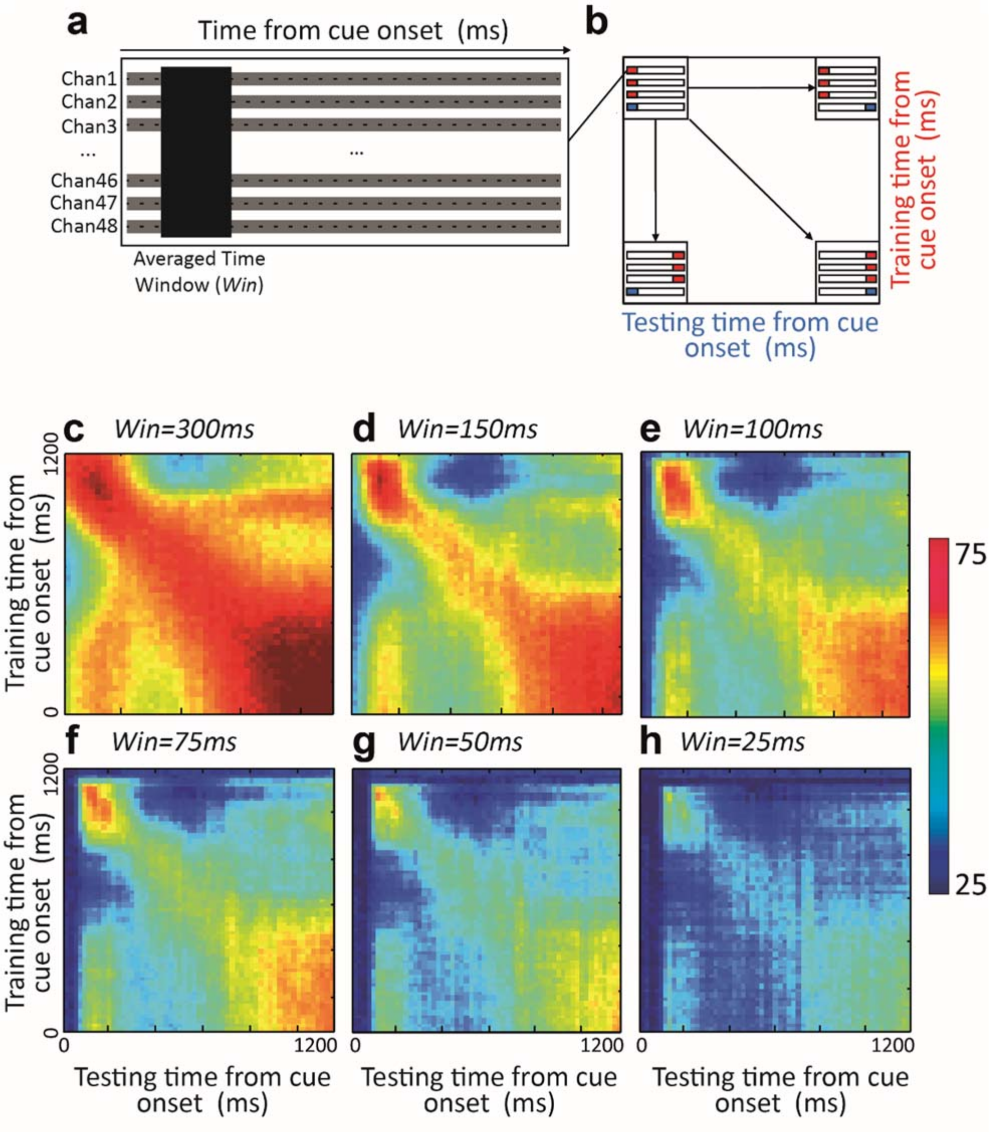

Supplementary Figure 3: **Cross temporal decoding and the impact of averaging time windows.** (a) Data structure on a given trial: MUA activity is recorded onto 48 channels, in time (1 ms resolution), aligned with respect to the cue, and averaged over time windows of length *Win*. (b) Cross-temporal decoding matrices are obtained by training a decoder on activities from the 48-channels, collected at a given time *t,* averaged over ATW ms, on a subset of trials (random 70%) and testing this decoder on activities from the 48-channels, collected, averaged over ATW ms, at all possible times (resolution of 10ms), on the remaining 30% test trials. This procedure is repeated over and over by moving reference time *t* by 10 ms each time, from 0 ms to 1200 ms from cue presentation. (c-h) Cross-temporal decoding matrices with different averaging time windows from 300 ms (c), to 150 ms (d), to 100 ms (e), to 75 ms (f) , to 50 ms (g) , to 25 ms (h) averaging window. 50 ms averaging windows reveals oscillations in the decoding performance along the testing time dimension (x-axis). These oscillations can already be seen at *Win*=75 ms.

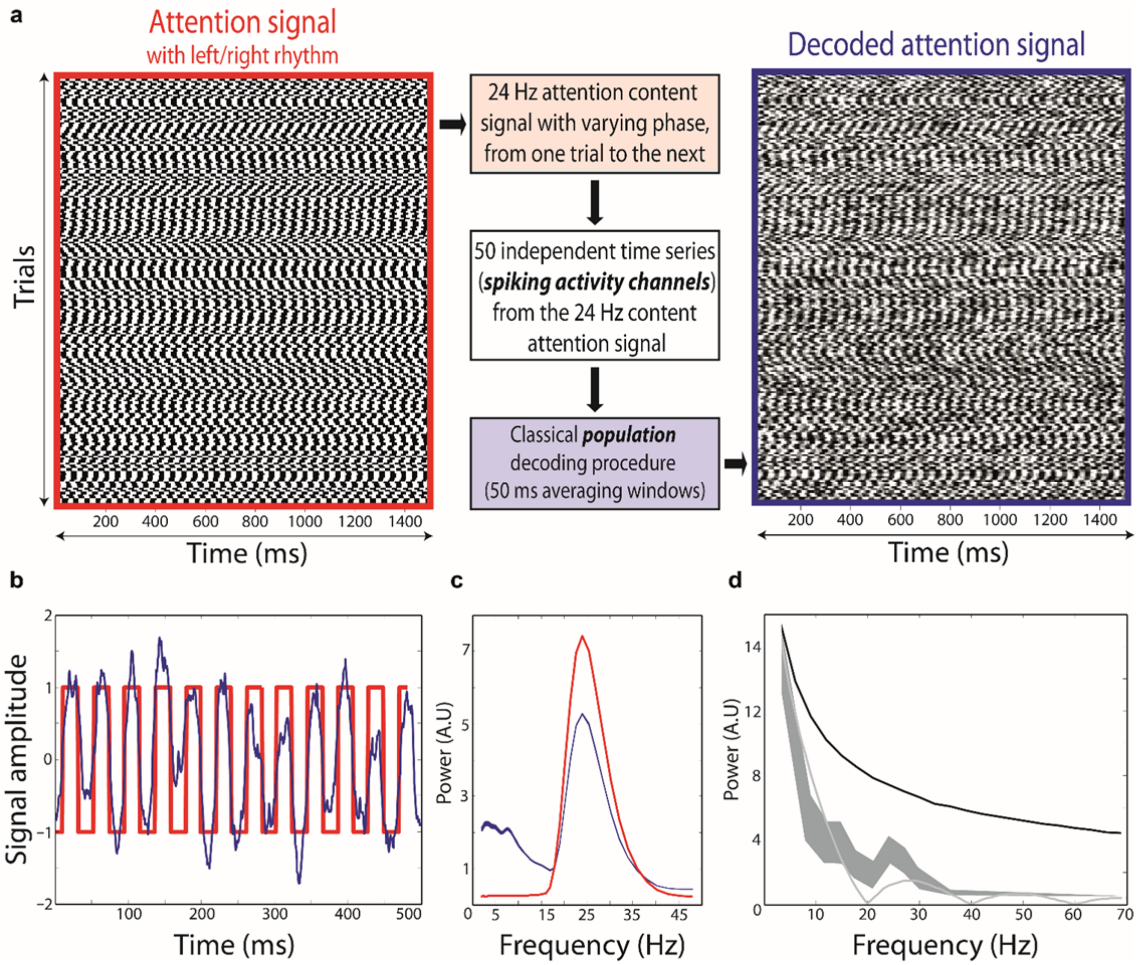

Supplementary Figure 4: ***Influence of moving average filtering procedure on decoded signal frequency content.*** (a) Artificial spatial attention signal exploring left/right space rhythmically at 24Hz, in time (x-axis), across multiple trials (y-axis), left panel. Fifty independent times series (representing the spiking activity of 50 independent MUA channels), are generated from the corresponding artificial attentional signal, each associated with a specific spiking probability that varied as a function of the artificial attention signal (position of attention). On each trial, the times series of these 50 channels are used for decoding the spatial position of attention in time (right panel). (b) Decoded spatial attention information (blue) and input rhythmic spatial attention signal (red), on the same representative trial. (c) Frequency analysis of decoded spatial attention time series (blue) and input rhythmic spatial attention time series (red). (d) Generalization of decoder attenuation profile on frequencies ranging from 3 to 70Hz (intermediate gray) compared to single channel attenuation profile after moving average filtering (fa=20Hz). Black: test frequency power relationship.

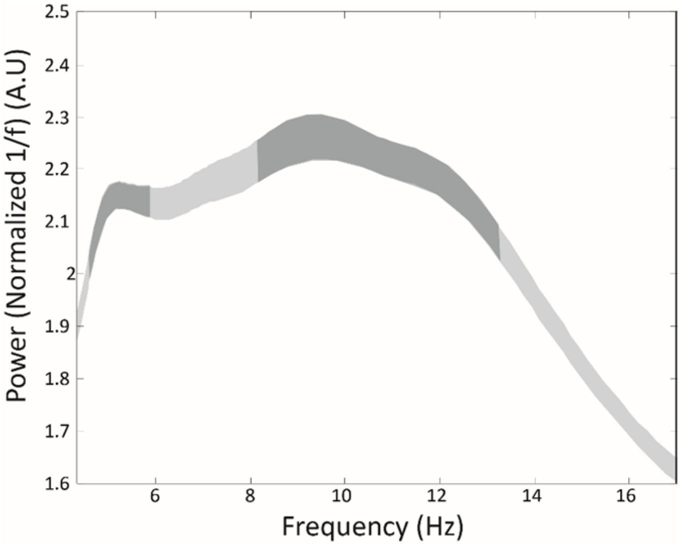

Supplementary Figure 5: ***Averaged peak oscillations of prefrontal attention-related information across all sessions.*** Normalized power identified in the cross-temporal classification reference interval (fig. 2b), (dark grey: significant frequencies relative to 95% C.I.) for all sessions (4-cued task version). Two significant local maxima can be identified against the 95% confidence interval, one in the theta range and one in the alpha range. The alpha peak is higher than the theta peak and coincides with the attentional rhythms discussed in the present work. Importantly, while the alpha attention information oscillation can be identified in all sessions (100%), the theta oscillation can only be identified in 68% of the sessions (13/19 sessions, significance assessed against the 95% confidence interval).

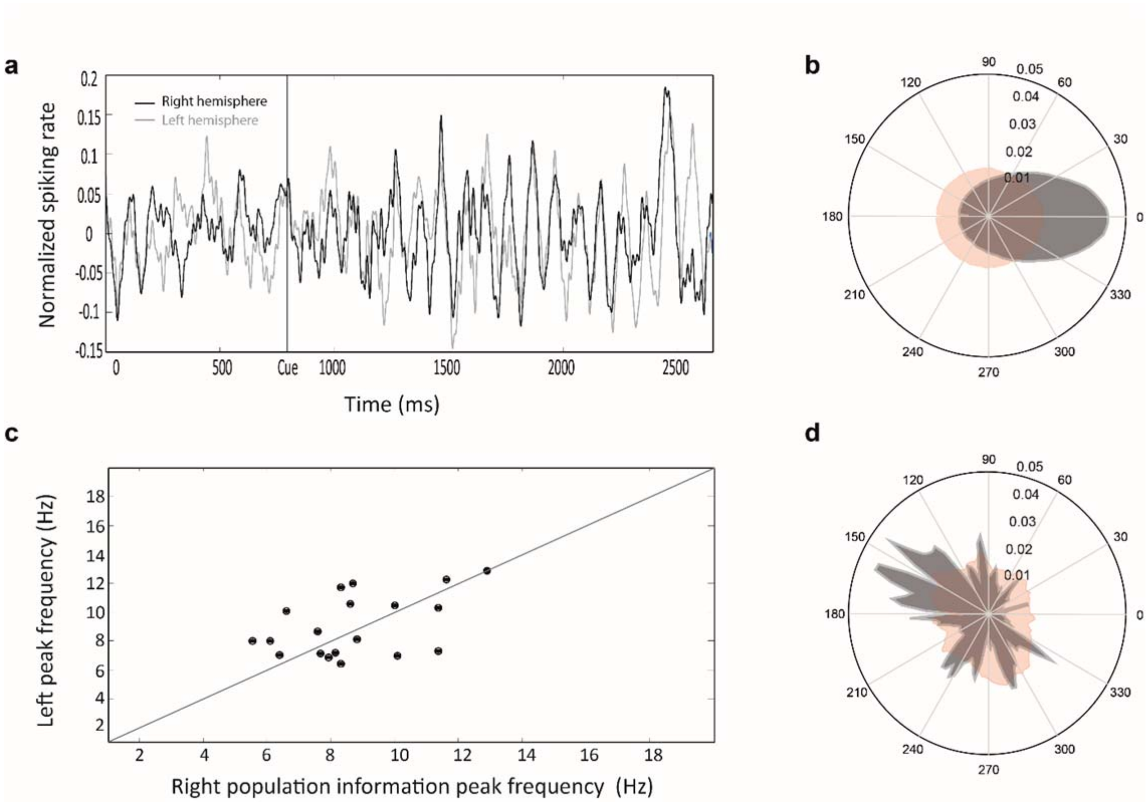

Supplementary Figure 6: ***Frequency and phase of attentional rhythm across hemispheres.*** (a) Single trial example of super MUA across left (grey) and right hemifield (black) recordings, on a representative session. (b) Distribution of phase relationship between the left and right super MUA alpha content, over all trials of all sessions (gray) and corresponding 95%CI (red). (c) Peak frequencies identified in the cross-temporal classification based on either right or left population information and (d) distribution of corresponding phase relationship (95%CI red).

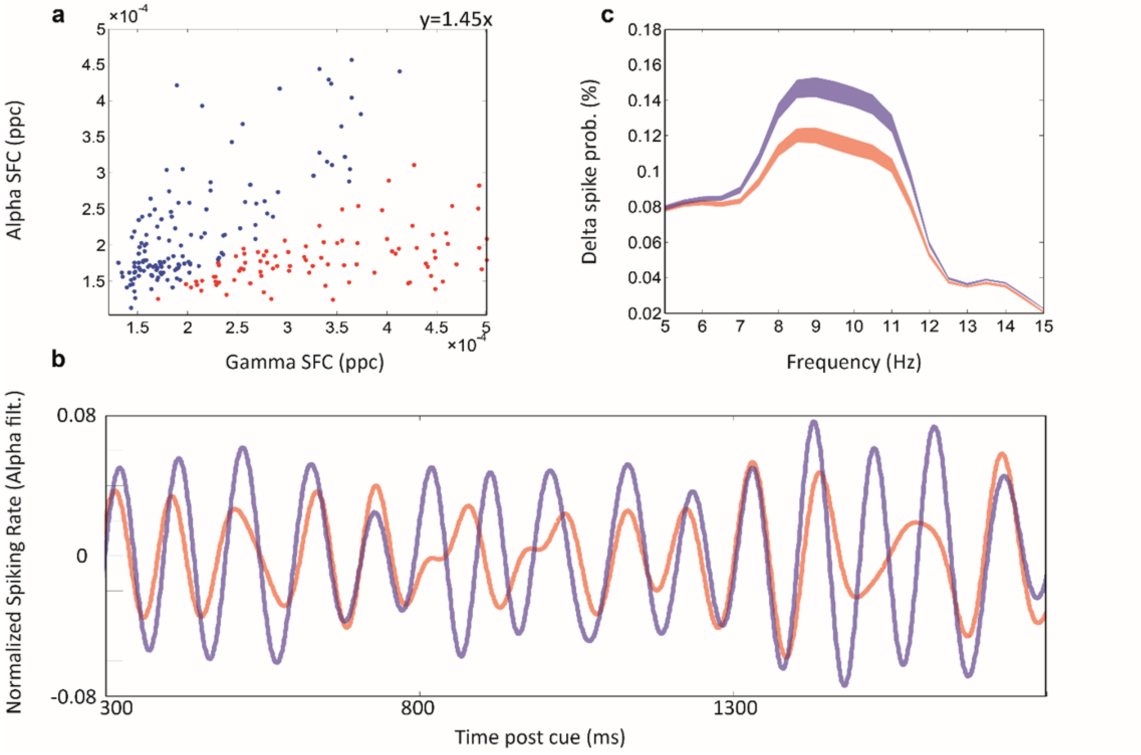

Supplementary Figure 7: ***Alpha spiking rhythm changes across prefrontal cortical layer*** (a) Spike Field Coherence based layer segregation defined by LFP alpha / gamma ratio (slope y=1.4x). (b) Alpha filtered super MUA activity across deep (blue) and superficial (red) recorded contacts for a representative trial. (c) Deep layer MUA contact present significantly higher alpha locking spiking activity.

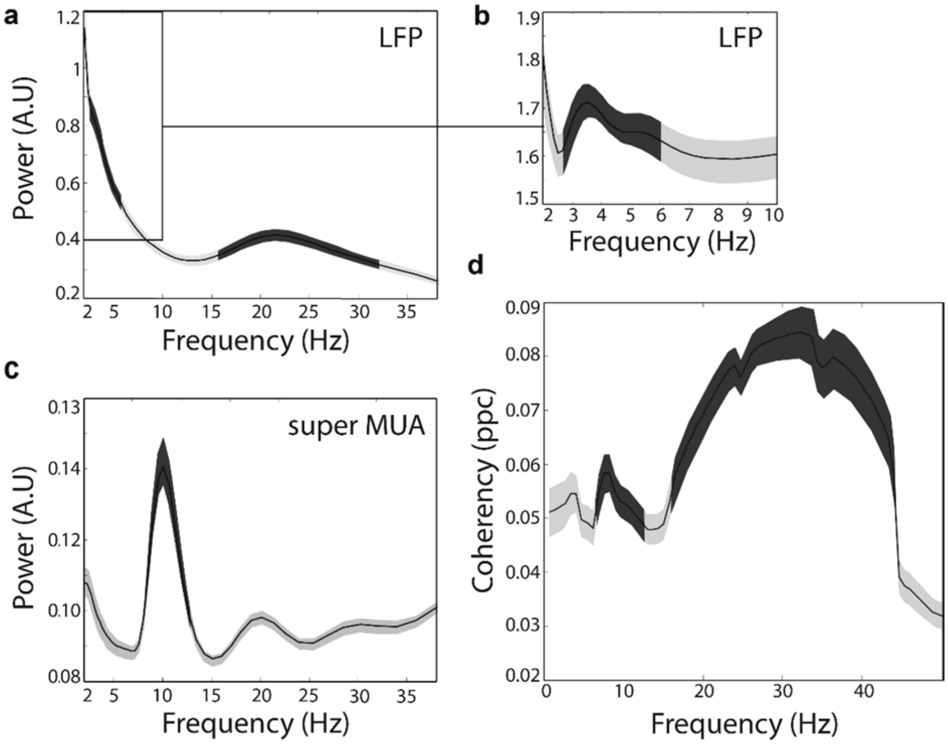

Supplementary Figure 8**: Alpha phase coherence between super MUAs and LFPs**. (a) Averaged LFP power spectrum (mean +/- s.e across all channels and sessions, dark grey: significance w/ 95%CI). (b) Close-up on low-frequency inset in (a), after 1/f correction. (c) Averaged super MUA power spectrum (mean +/- s.e, across all channels and sessions, dark grey: significance w/ 95%CI). (d) Averaged phase coherency between super MUAs and LFPs, calculated using pairwise phase consistency (PPC) (mean +/- s.e, across all channels and sessions, dark grey: significance w/ 95%CI).
