# Supplementary material for: Prefrontal attentional saccades explore space rhythmically
Source: Nat Commun. 2020 Feb 17;11:925. doi: 10.1038/s41467-020-14649-7 (PMC7026397; doi:10.1038/s41467-020-14649-7)
Supplement: Supplementary file 4 — Description of Additional Supplementary Files [file 41467_2020_14649_MOESM4_ESM.pdf]

Description of Additional Supplementary Files

File Name: Supplementary Movie 1

Description: Real-time space exploration by decoded prefrontal attentional spotlight during an exemplar trial (white trace, fading represents memory of attentional trace at prior locations). Time runs from 500 ms before cue presentation to 1500ms following cue presentation. Gray squares: stable task landmarks; Red peri-central square: cue. Fixation point at the center of the screen not displayed. Eye traces not displayed (eye fixations 1° away from the fixation point interrupted the trial).
